# Generating Angular-Varying Time Delays of THz Pulses via Direct Space-to-Time Mapping of Metasurface Structures

**DOI:** 10.1021/acsaom.3c00240

**Published:** 2023-10-30

**Authors:** Elazar Elias, Symeon Sideris, Cormac McDonnell, Tal Ellenbogen

**Affiliations:** † Raymond and Beverly Sackler School of Physics and Astronomy, Tel Aviv University, Ramat Aviv, Tel Aviv 6779801, Israel; ‡ Center for Light-Matter Interaction, Tel-Aviv University, Tel Aviv 6779801, Israel; § Department of Physical Electronics, School of Electrical Engineering, Tel-Aviv University, Tel Aviv 6997801, Israel

**Keywords:** Terahertz, wavefront shaping, space to time mapping, double pulse generation, pump−probe experiments, Pancharatnam-Berry geometric phase

## Abstract

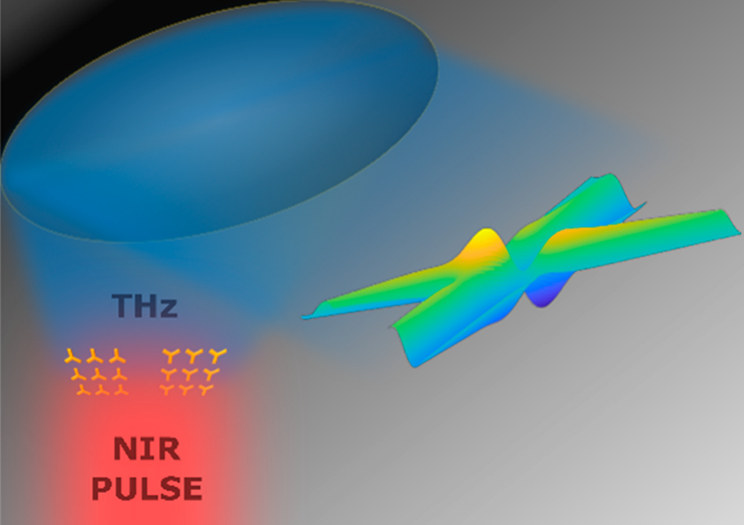

We experimentally demonstrate the generation of double terahertz (THz) pulses with tailored angular-dependent time delays from a nonlinear metasurface excited by a near-infrared femtosecond pulse. The tailored temporal properties of the generated pulses emerge from a direct mapping of the nonlinear spatial response of the metasurface to the emitted THz temporal profile. We utilize the Pancharatnam-Berry phase to implement symmetric and antisymmetric metasurface configurations and show that the emitted patterns present spatiotemporal “X-shaped” profiles after collimation by a parabolic mirror, with angular-dependent pulse delays corresponding to the intended design. In addition, we show that the addition of polarization multiplexing presents the opportunity to achieve a full range of elliptical THz polarizations. Double pulse generation and spatiotemporal shaping of THz waves in general show potential for THz spectroscopy and molecular dynamics applications, particularly in pump–probe experiments.

## Introduction

The terahertz (THz) band refers to the region of the electromagnetic spectrum that lies between the well-developed microwave and optical spectral ranges. While the technological challenges of generating and detecting radiation in this electromagnetic region resulted in adopting the term “THz gap”, research has revealed the significance of exploring THz frequencies for various applications. Many materials exhibit vibrational and rotational transitions in the THz frequencies, making THz spectroscopy an important tool for studying fundamental physical and chemical interactions,^1−3^ along with practical applications such as material identification.^4−6^ Moreover, by shaping the temporal profile of the THz pulses, it is possible to align gas molecules in air, which opens the door not only for studying but also for controlling molecular dynamics.^7^ Furthermore, THz detectable differences of water content in tissues were shown to be useful for biomedical imaging,^8,9^ which is highly attractive for constant monitoring needs due to the low energies of THz radiation (∼meV) that do not harm biological tissues, as opposed to X-rays (with energies of ∼keV). In addition, a great effort is put into utilizing the high carrier frequencies of the THz band which promise large data rates and channel capacities for next-generation communications.^10−13^ These applications can be further improved through optimization of the spatial and temporal properties of THz waves. However, due to various challenges, such as material absorption in this spectral region, creating broadband optical elements is not straightforward, and innovative beam-shaping methods have been investigated. For example, enhanced contrast and edge detection of THz imaging were recently achieved using spiral spatial filtering;^14^ nondiffracting THz Bessel waves were realized and demonstrated improved image resolution and a spatiotemporal coupling with an X-shape profile;^15,16^ and single-cycle THz vortex beams were generated and used to investigate nonlinear absorption in bilayer graphene.^17^ In addition, THz pulsed time domain holography (THz-PTDH), a technique for high resolution imaging of THz amplitude and phase, has been introduced and explored,^18−20^ allowing mapping of spectroscopic information in the imaged object. Improving the ability of complex spatial and temporal wavefront shaping of THz radiation, along with utilizing the THz-PTDH technique, may further improve these emerging THz technologies.

Throughout the years, several methods and techniques were developed for generating THz radiation, including photoconductive antennas,^21^ down conversion in nonlinear crystals,^22^ spintronic emitters,^23^ quantum cascade lasers,^24,25^ and free electron lasers.^26^ While these sources can often emit at high output powers, it can be challenging to shape the THz beam post output, and several methods have been explored.^27^ One promising direction to obtain better control over the properties of THz waves is using metasurfaces, which allow the simultaneous generation and shaping of radiation. Over the past decade, optical metasurfaces have shown a huge array of applications in the generation and control of light. These nano-engineered devices consist of either metallic or dielectric subwavelength building blocks structured in a single thin layer. Their subsequent interaction with an incident wave can result in a wide range of designed functionalities. For example, metasurfaces were used to apply phase gradients and phase discontinuities,^28^ resulting in optical beam shaping and beam steering and enabling the design of metalenses.^29−31^ In addition, polarization conversion, optical holography, controlled frequency conversion, and harmonic generation were realized using metasurfaces.^32−34^ Recently, nonlinear processes such as broadband THz generation have also been demonstrated using nonlinear metasurfaces (NLMSs).^35^ Gold split-ring resonators were shown to generate broadband single-cycle THz radiation when pumped with femtosecond near-infrared (NIR) pulses.^36^ Moreover, 3-fold rotationally symmetric (C3) meta-atoms were studied and showed the ability to continuously control the polarization state of the emitted THz through simple rotations of the linear polarization angle of the pump, owing to the accumulated Pancharatnam-Berry (P–B) phase.^37^ In addition, the theoretical concept of direct space-to-time (DST) mapping has been recently introduced,^38^ which enables the nonlinear spatial response profile of the metasurface to be directly mapped to the THz temporal profile in the far field. Along with the versatility in THz polarization rotation that the C3 meta-atoms allow, the DST mapping enables the design of desired spatiotemporal THz profiles, presenting crucial development in THz wavefront shaping.

This work experimentally demonstrates the DST mapping principle using C3 gold meta-atoms for the tailored generation of double THz pulses with varying time delays. We spatially control the temporal delay between two generated single-cycle THz pulses and use the P–B phase of the C3 shape to generate pulses with similar or opposite polarity, resulting in different X-shaped far-field spatiotemporal interference patterns of the emitted THz radiation. We then explore polarization multiplexing and show the potential for generating angular-varying THz polarization states. The generation of two pulses with varying time delays opens the door to various pump–probe studies and the study of molecular dynamics.

## Results

**Figure 1:**
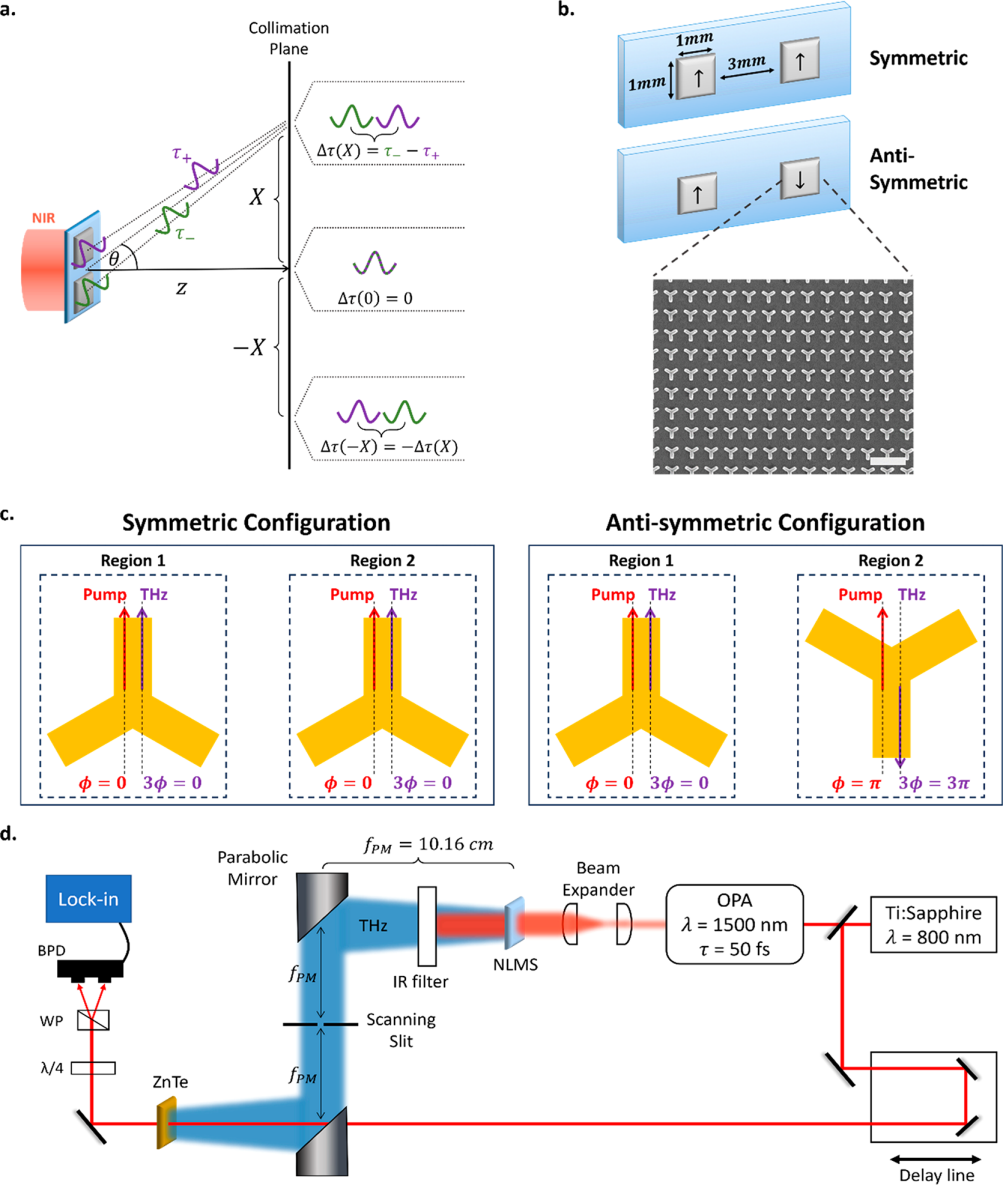
(a) Schematic illustration showing the space-to-time mapping of the metasurface's nonlinear spatial response structure to the far-field temporal profile of the emitted THz waves. (b) Schematic illustration of the samples, consisting of symmetric and antisymmetric configurations, each realized by two 1 mm × 1 mm uniform regions. The arrows indicate the meta-atoms are oriented either up or down in each uniform region. The inset shows a scanning electron microscope image of a fabricated uniform region of the metasurface consisting of C3-symmetric gold meta-atoms. The arms of each meta-atom are 200 nm long and 80 nm wide, and the square lattice of the meta-atoms in each uniform region has a periodicity of 550 nm. Scale-bar is 1 μm. (c) Symmetric and antisymmetric configurations and their respective pump and THz emission polarities, resulting from the P–B phase properties of the C3 meta-atoms. (d) THz-TDS setup used for generation and detection of the THz pulses from the metasurface. The NLMS was located at the focus of the collimating parabolic mirror, *f*_PM_ = 10.16 cm, and the scanning slit was located in the center of the collimated space, *f*_PM_ from each mirror.

The direct space-to-time mapping concept implies that under excitation of a nonlinear metasurface at normal incidence the spatial nonlinear function that is printed on the metasurface translates to the temporal function of the THz waveform at the far field. Mathematically, the electric field observed at a transverse location *X* in the far field, corresponding to an angle θ at distance *z* from the metasurface, can be represented by the convolution:^38^
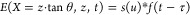
1where *s*(*u*) is the spatial profile of the nonlinear function of the metasurface, and the transverse coordinate *u* on the metasurface is directly mapped to time according to *t* = *u*·sin θ/*c*, where *c* is the speed of light. *f*(*t*) is the temporal profile of the kernel pulse and is delayed by
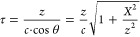
2It was shown theoretically that this concept can be used to tailor complex THz waveforms.^38^ However, the concept can also be demonstrated by a simple system of double pulse generation, as shown in Figure 1a. Considering two pulses, one originating closer to the observer at a transverse location +*d*/2 on the metasurface with an arrival time of τ_+_ and another pulse originating further from the observer at −*d*/2 with arrival time τ_–_, the delay between the two pulses at the far field is
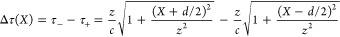
3It can be seen that for an observer located at *X* there will be a delay between the two pulses, while for an observer located at *X* = 0 the pulses will arrive simultaneously. For an observer located at −*X*, the time separation between the pulses will be the same as for an observer located at *X*, but as shown in Figure 1a, τ_–_ will arrive ahead of τ_+_, meaning Δτ(−*X*) = −Δτ(*X*).

To experimentally verify this concept, we examined the generation and propagation of THz waves from different sample configurations. We fabricated symmetric and antisymmetric double-pulse generators as shown schematically in Figure 1b. All the regions of the metasurface consist of gold meta-atoms with C3 rotational symmetry grown on a glass substrate coated with 20 nm thick ITO film, where the ITO surrounding the meta-atoms was etched in the last step of the fabrication process in order to increase the radiation efficiency of the emitters.^39^ As was previously shown,^37^ each of these uniform regions generates single-cycle broadband THz radiation upon incidence with a femtosecond NIR pulse. In addition, due to an accumulated P–B phase, for any pump polarization angle ϕ relative to the principal axis of the meta-atom, the generated THz polarization angle is rotated by 3ϕ relative to the pump. This results in an all-optically controlled phase and polarization state of the emitted THz beam. Specifically, as presented in Figure 1c, pumping the meta-atom along its principal axis results in THz polarized along this axis. If the orientation of the meta-atom is inverted relative to the pump, the generated THz is inverted accordingly, resulting in an opposite phase compared to the THz generated from a noninverted meta-atom. One pair of regions was fabricated with both regions having the same orientations of the meta-atoms and is thus termed the “symmetric” pair, while the regions of the other pair were fabricated with reversed orientations relative to one another and hence are termed the “antisymmetric” pair, as schematically shown in Figure 1c. We note that each uniform region consisted of meta-atoms oriented in the same direction and the only difference in orientations was between the two different regions of the antisymmetric configuration.

For both pairs, each uniform region is 1 mm × 1 mm and the separation between the regions is 3 mm. These design parameters were chosen to maximize the collection of the generated THz waves into the numerical aperture of the collection optics. A vertically polarized ultrashort laser with pulse duration of ∼50 fs centered at wavelength of ∼1500 nm was used to illuminate each pair, and the vertically polarized THz waves were measured using a THz time-domain spectroscopy (TDS) setup based on electro-optic (EO) sampling, as depicted in Figure 1d. A 0.5 mm thick ZnTe nonlinear crystal was used for EO sampling, providing a measurement bandwidth up to 2.5 THz. The metasurface was located at the focus *f*_PM_ = 10.16 cm of a collimating parabolic mirror. The parabolic phase imposed by the mirror and the free-space propagator achieve an approximate Fourier transform one focal distance after the mirror,^40^ and the emission angles are mapped into lateral translations in the collimated space. A 7 mm wide and 19 cm tall slit located in the center of the collimated space between the two off-axis parabolic mirrors, at *f*_PM_ from each mirror, was used to spatially filter and raster scan the transverse profile of the emitted THz waves. Considering the metasurface emitter as two sources in the momentum plane results in a Fourier pattern of two waves propagating at oblique angles relative to the optical axis. However, considering the temporal profiles of the generated THz waves are pulses and not continuous waves, the overall spatiotemporal profile is more complex.

**Figure 2:**
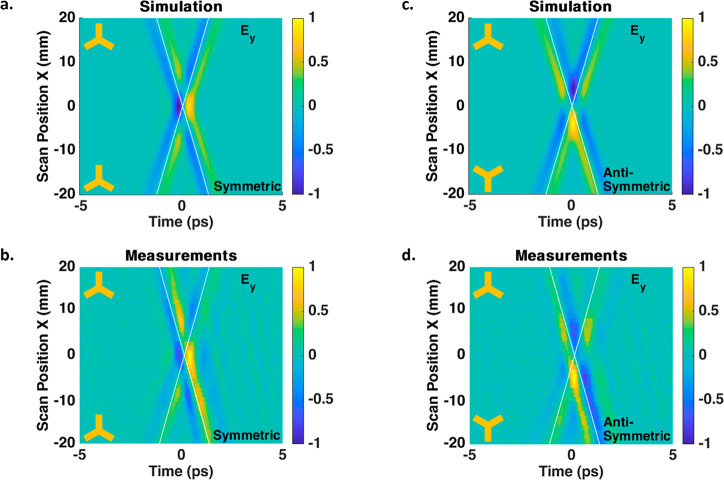
(a) Simulated and (b) measured normalized spatiotemporal profiles of the symmetric configuration; both present two same-phased single-cycle fronts propagating with angular-varying time delays, showing constructive interference in the center. The profiles are presented in the collimated space, and the white diagonal lines represent the centers of the pulses. (c) Simulated and (d) measured normalized spatiotemporal profiles of the antisymmetric configuration, showing destructive interference in the center. The C3 shapes show schematically the orientations of the two uniform regions in each configuration.

To obtain a better understanding of the expected emission patterns, we performed beam propagation simulations (see Experimental Section). The normalized simulated and measured spatiotemporal THz profiles of the symmetric configuration are presented in Figures 2a,b, respectively. There is excellent agreement between the experiment and simulation, both showing two single-cycle THz pulses with the same phase and having different time delays for different transverse locations. The position-dependent phase shift induced by the parabolic mirror collimates the beams by compensating for the curvatures of the two spherical wavefronts emitted from the two regions of the metasurface, and forms an X-shaped spatiotemporal profile. The central positions of the pulses calculated from the space-to-time mapping are represented by the white diagonal lines, which were obtained by considering the arrival times τ_+_ and τ_–_ presented in eq 3, shifted by  as this is the arrival time from the center of the metasurface.

We notice constructive interference between the pulses is present in the center of the profile, as expected for pulses with the same phase and no time delay. Continuous increase of the time delay between the two pulses is achieved for larger lateral positions, up to ∼2.5 ps time delay for *X* = 20 mm in the collimated space, providing δτ/δ*X* = 0.125 ps/mm. This value can be controlled by the separation between the generating regions, according to eq 3. Figures 2c,d show the normalized simulated and measured spatiotemporal profiles of the antisymmetric configuration, respectively, and are also in good agreement, showing destructive interference in the center of the profile. We note that while the measurements were achieved using a slit and not an aperture, meaning the THz signal was integrated over the *y*-axis, the Fourier transform of two emitters separated in the *x*-axis is approximately independent of the *y* direction, and hence, the integrated signal is a good representation of a 1D signal.

**Figure 3:**
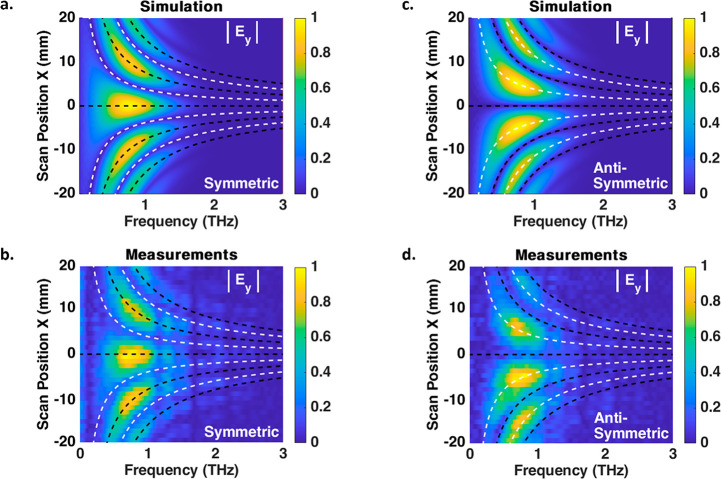
(a) Simulated and (b) measured normalized spatio-spectral amplitude profiles of the symmetric configuration show good agreement and present several THz diffraction orders (black dashed lines), including the dominant zeroth-order due to constructive interference of the same-phased pulses. (c) Simulated and (d) measured normalized spatio-spectral amplitude profiles of the antisymmetric configuration. Several diffraction orders are present (white dashed lines) with no zeroth-order due to destructive interference of the signals from the two oppositely oriented regions of the antisymmetric configuration.

The corresponding normalized simulated and measured spatio-spectral amplitude profiles are presented in Figure 3. The central THz frequency is around 0.8 THz, and several diffraction orders that match the classic interference pattern from two slits can be observed. We can see the spectral dependence on the temporal separation between the pulses. For increasing lateral scan positions, corresponding to larger diffraction angles, there is an increase in the separation between the two pulses and consequently an increase in the number of diffraction orders appearing in the spectrum. The black and white dashed lines represent the diffraction orders of the symmetric and antisymmetric configurations, respectively. We notice the peaks of the symmetric configuration are the zeros of the antisymmetric configuration and vice versa. Specifically, while the zeroth-order of diffraction is present for the symmetric configuration, as seen in Figure 3b, there is no THz illumination to the zeroth-order for the antisymmetric configuration in Figure 3d due to destructive interference between the oppositely phased pulses.

To show the potential applications of the generated spatiotemporal X-shaped profile in time delay pump–probe experiments, we performed Gaussian image filtering in order to reduce the experimental noise and examined the propagating THz pulses. It is expected that, at different time delays, the investigated system will go from constructive to destructive excitation due to the differing interactions of the double pulses. We simulated the spatiotemporal profiles with addition of a free induction decay (FID) signal due to the interaction of the THz waves with molecules in the air (see Experimental Section). The THz waves rotate the gas molecules in the air, and as they periodically co-orient, they coherently emit FID signals.^3^

**Figure 4:**
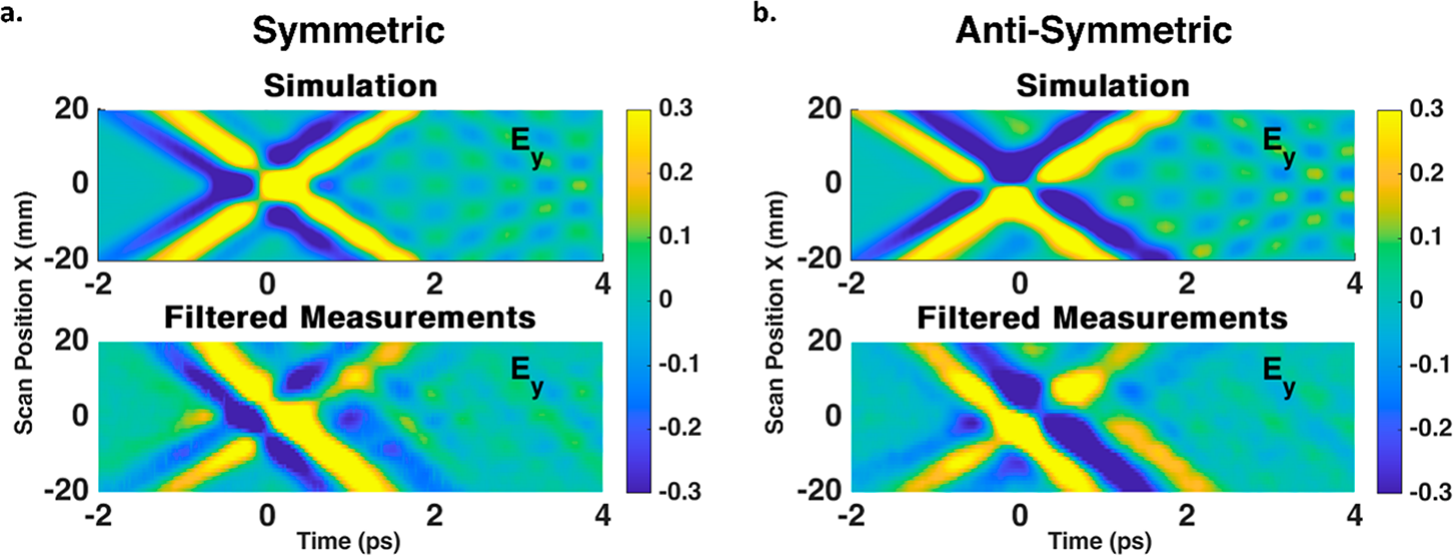
Comparison of the saturated (a) symmetric and (b) antisymmetric normalized spatiotemporal profiles between simulations including FID emission (top) and saturated measurements after applying Gaussian image filtering to the raw data (bottom). For both configurations, the measurements exhibit interference patterns after the two main pulses which match those presented in the simulations, verifying the symmetric and antisymmetric nature of the THz profile and its ability to interact with gas molecules in air.

Figures 4a,b present the saturated normalized spatiotemporal profiles of the Gaussian-filtered symmetric and antisymmetric configurations, respectively, compared to the simulated profiles with the FID emission. The measured profiles show small interference features following the two main pulses. These interference patterns correspond to the symmetric and antisymmetric origins of the THz pulses, as seen in comparison to one another and in accordance with the simulated emission profiles. While the initial spatiotemporal X-shape is directly generated from the metasurface, the existence of these small interference patterns shows the system interacts with the generated THz pulses according to the designed temporal profile. The interference of such double pulses in time can be potentially applied to interact with ensembles of gas molecules in air, controlling their rotational transitions and thus emitting coherent THz radiation via FID, as the simulations suggest. The spatial dependency of the interpulse delay may allow the study of these excitations for a wide range of different time delays using one-dimensional raster scanning, without the need to fabricate different samples or undertake time intensive realignment of the measurement setup. This can prove useful for pump–probe experiments, where the initial pulse pumps the gas molecules and the second pulse examines the dynamics.

**Figure 5:**
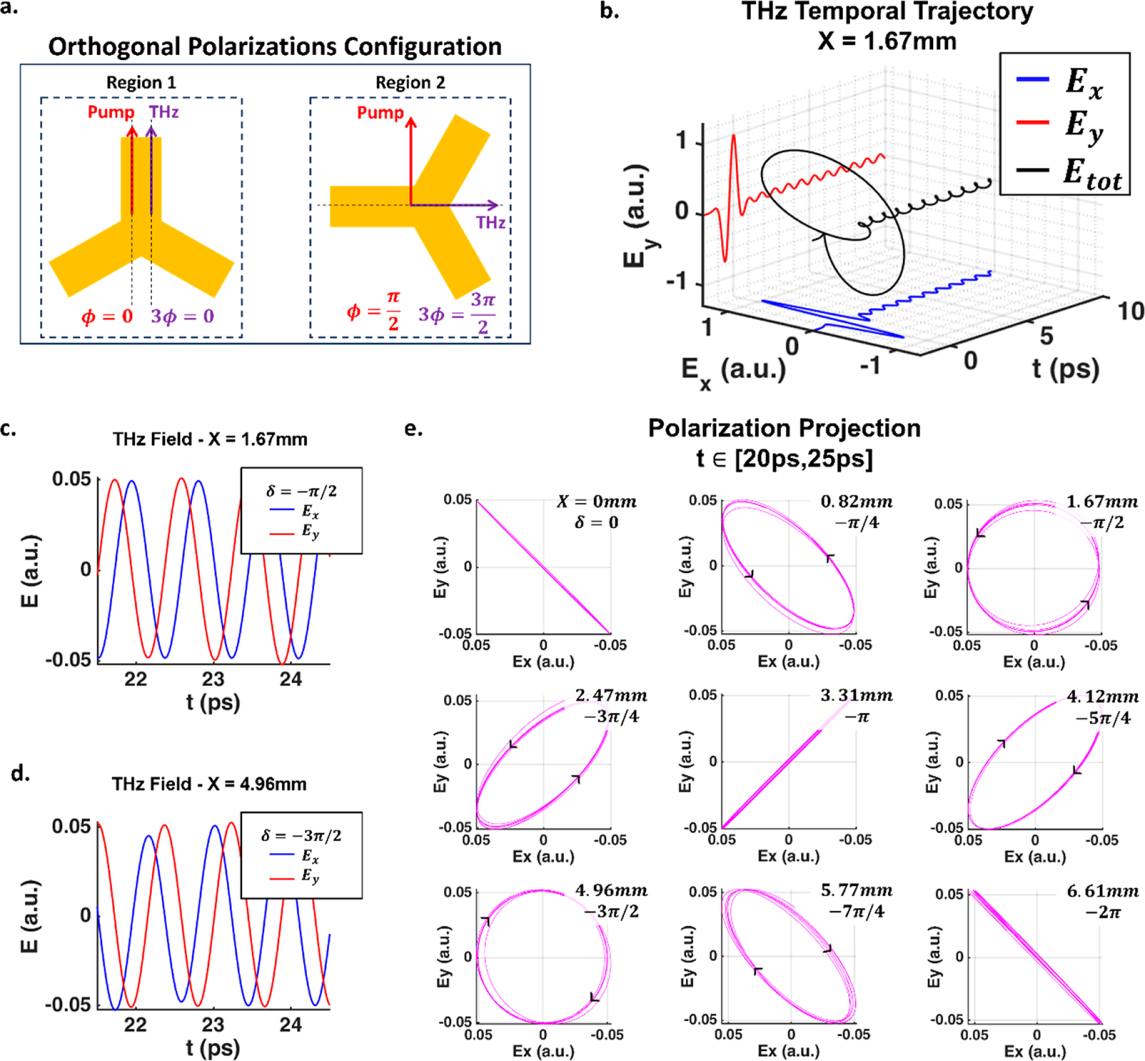
(a) Suggested orthogonal double-pulse generator configuration of the metasurface, where the meta-atoms in one region are oriented perpendicular to the meta-atoms in the other region, thus emitting orthogonally polarized THz pulses. (b) Temporal trajectories of the total simulated THz field (black) and its *E*_*x*_ (blue) and *E*_*y*_ (red) components at *X* = 1.67 mm. Temporal separation between *E*_*x*_ and *E*_*y*_ results in a rotated field trajectory and ellipticity in polarization. (c) Simulated temporal profiles of *E*_*x*_ and *E*_*y*_ components at *X* = 1.67 mm show *E*_*x*_ preceding *E*_*y*_ with phase difference δ = −π/2, corresponding to LCP state. (d) Simulated temporal profiles of *E*_*x*_ and *E*_*y*_ components at *X* = 4.96 mm show *E*_*y*_ preceding *E*_*x*_, with phase difference δ = −3π/2, corresponding to RCP state. (e) Polarization projections of the total simulated THz field in the *E*_*x*_–*E*_*y*_ plane for different lateral positions present a full 2π range of phase differences between the field components, resulting in different THz polarization states.

We now utilize the P–B phase presented by the C3 meta-atoms to simulate a complex THz profile comprising both *x̂* and *ŷ* linear polarizations. By rotating one region of the metasurface perpendicular to the other, we can generate both linear polarizations, each from a different region, as depicted in Figure 5a. We expect that, for different lateral positions, which correspond to different time delays, there will be a change in the phase difference δ = φ_*y*_ – φ_*x*_ between the electric field components with φ_*x*_ and φ_*y*_ being the phases of the *E*_*x*_ and *E*_*y*_ components of the total THz field, respectively. In our simulations, we used a simple scalar approach in which the THz pulses emitted from each uniform region were treated separately due to the C3 polarization rotation, which implies the emission of orthogonal polarizations, and hence, the signals from the two regions do not interfere. A more general approach involving Jones matrices formalism for THz radiation propagation and its interaction with beam-shaping devices was discussed elsewhere.^41^

We focus on the FID signal present in the tail of the profile, for which the phase difference is better defined. Figure 5b shows the simulated temporal trajectory of the total emitted THz field and FID signal for *X* = 1.67 mm, along with its *E*_*x*_ and *E*_*y*_ components. The temporal delay and phase difference between the orthogonal components, caused by the DST mapping, result in a rotated trajectory of the total electric field. Figures 5c,d show the simulated temporal profiles of *E*_*x*_ and *E*_*y*_ components for *X* = 1.67 mm and *X* = 4.96 mm, respectively, where the time separation between the field components is ∼1/4 of the cycle period. For *X* = 1.67 mm, *E*_*x*_ precedes *E*_*y*_, resulting in the phase difference δ = −π/2, which corresponds to a left circular polarization (LCP) state. For *X* = 4.96 mm, *E*_*y*_ precedes *E*_*x*_ and thus δ = −3π/2, corresponding to a right circular polarization (RCP) state. Figure 5e presents the time trajectory projections (from 20 ps to 25 ps) on the *E*_*x*_–*E*_*y*_ plane for different lateral scan positions. We see the trajectory projection of the total THz field changes from linear −45° polarization at 0 mm to LCP at 1.67 mm, to the orthogonal linear 45° polarization at 3.31 mm, RCP at 4.96 mm, and back to linear −45° polarization at 6.61 mm, thus completing the full 2π change of the phase difference between the field components. Different elliptical polarizations are also presented along this phase sweep. We note that the polarization projections shown in Figure 5e present slight deviations from perfect polarization trajectories due to differences in field amplitudes of *E*_*x*_ and *E*_*y*_, caused by the DST mapping for increasing lateral positions. These slight amplitude differences can also be seen in Figures 5c,d.

## Conclusions

## Experimental Section

### Fabrication

The metasurfaces under study were fabricated on commercially available ITO-coated glass substrates. Initially, the substrates were placed in an acetone solution and cleaned using sonification. The substrates were then dried under a stream of N_2_, followed by the deposition of a thin film of a positive electron resist (PMMA), which was spin coated on top of the ITO-coated glass. The PMMA layer was then baked at 180 °C for 1 min, and the metasurfaces were written using a standard electron beam lithography (EBL) system. After the development of the samples, 40 nm of Au followed by 25 nm of Cr were deposited on top of the photoresist using an electron beam evaporator. The remaining photoresist was lifted off by submerging the metasurfaces in acetone. In the next step, the ITO layer surrounding the metasurfaces was removed by using reactive ion etching (RIE). The RIE process was performed in a gas mixture of CHF3 and Ar with gas flow rates of 40 ccm and 10 ccm, respectively, while the gas mixture was excited with 250 W. The chamber’s pressure was kept at 40 mTorr, and the etching time was set to 3 min. To remove the Cr layer, the sample was deposited in a commercial Cr etchant solution, followed by an additional immersion in a deionized water solution for 1 min and dried under a N_2_ stream.

### THz Time Domain Spectroscopy

A Spectra-Physics Solstice Ace femtosecond laser source was used to generate ∼35 fs pulses at 800 nm with a 2 kHz repetition rate and 3.5 mJ per pulse. The output was split into the pump and probe lines. In the pump line, it was converted to an ∼50 fs pump centered at 1500 nm using a TOPAS optical parametric amplifier. A half waveplate and a polarizer were used to control the laser power along with a mechanical chopper operating at 1 kHz. Two cylindrical lenses were used to change the spot shape from a circular to a horizontally oriented elliptical spot to excite the 2 regions of the metasurface. The metasurface was located at the focal point of an off-axis parabolic mirror (*f*_PM_ = 10.16 cm) to collect and collimate the generated THz. A 5 mm thick Teflon slab was placed between the metasurface and the parabolic mirror to filter out the pump beam. A 7 mm wide and 19 cm tall slit was mounted on a moving stage to raster scan the THz signal horizontally in the collimated space, before another off-axis parabolic mirror (*f*_PM_ = 10.16 cm) was used to focus the THz into a 0.5 mm ⟨110⟩ cut ZnTe nonlinear crystal for electro-optic detection. In the probe line, a small portion of the 800 nm source was sent to a motorized delay stage, which is used to control the temporal overlap between the generated THz and the probe in the ZnTe crystal, with the probe being focused into the crystal after being directed through a 3 mm hole in the second parabolic mirror. After passing through the crystal, the THz-induced electro-optical effect on the probe was measured by a set composed of a collimating lens, a quarter-wave plate, a Wollaston prism, and a balanced photodiode. The signal from the photodiode was amplified using a Standford Research Systems SR830 lock-in detector, locked to the 1 kHz frequency of the mechanical chopper, synchronized to a subharmonic of the laser system.

### THz Beam Propagation Simulations

Wave propagation simulations were implemented using MATLAB. The broadband pulse was defined by a spectrum that matched the measured THZ spectrum from a single uniform NLMS. The electric field generated from the regions of the NLMS was defined for each frequency to have either positive or negative phase and was set to zero outside the regions. The spatial Fourier components were propagated along the *z* direction separately for each temporal frequency, and the collimation of the beam by a parabolic mirror was simulated by using an additional phase factor. The spatiotemporal profiles were achieved via an inverse Fourier transform in time and space. FID signals were simulated by sine waves of frequencies 1.16 THz, 1.44 THz, and 1.69 THz, corresponding to absorption lines in the measured spectra, with 0 or π phase according to the symmetry of the configuration. These sine waves were simulated to be emitted from the centers of the propagating pulse fronts.
